# Direct In Situ Growth of Centimeter‐Scale Multi‐Heterojunction MoS_2_/WS_2_/WSe_2_ Thin‐Film Catalyst for Photo‐Electrochemical Hydrogen Evolution

**DOI:** 10.1002/advs.201900301

**Published:** 2019-04-26

**Authors:** Sehun Seo, Seungkyu Kim, Hojoong Choi, Jongmin Lee, Hongji Yoon, Guangxia Piao, Jun‐Cheol Park, Yoonsung Jung, Jaesun Song, Sang Yun Jeong, Hyunwoong Park, Sanghan Lee

**Affiliations:** ^1^ School of Materials Science and Engineering Gwangju Institute of Science and Technology Gwangju 61005 Republic of Korea; ^2^ School of Energy Engineering Kyungpook National University Daegu 41566 Republic of Korea

**Keywords:** heterostructures, hydrogen evolution, photo‐electrochemical, pulsed laser deposition, transition metal dichalcogenides

## Abstract

To date, the in situ fabrication of the large‐scale van der Waals multi‐heterojunction transition metal dichalcogenides (multi‐TMDs) is significantly challenging using conventional deposition methods. In this study, vertically stacked centimeter‐scale multi‐TMD (MoS_2_/WS_2_/WSe_2_ and MoS_2_/WSe_2_) thin films are successfully fabricated via sequential pulsed laser deposition (PLD), which is an in situ growth process. The fabricated MoS_2_/WS_2_/WSe_2_ thin film on p‐type silicon (p‐Si) substrate is designed to form multistaggered gaps (type‐II band structure) with p‐Si, and this film exhibits excellent spatial and thickness uniformity, which is verified by Raman spectroscopy. Among various application fields, MoS_2_/WS_2_/WSe_2_ is applied to the thin‐film catalyst of a p‐Si photocathode, to effectively transfer the photogenerated electrons from p‐Si to the electrolyte in the photo‐electrochemical (PEC) hydrogen evolution. From a comparison between the PEC performances of the homostructure TMDs (homo‐TMDs)/p‐Si and multi‐TMDs/p‐Si, it is demonstrated that the multistaggered gap of multi‐TMDs/p‐Si improves the PEC performance significantly more than the homo‐TMDs/p‐Si and bare p‐Si by effective charge transfer. The new in situ growth process for the fabrication of multi‐TMD thin films offers a novel and innovative method for the application of multi‐TMD thin films to various fields.

## 1 Introduction

The heterostructure based on atomically thin 2D transition metal dichalcogenides (TMDs) is a novel building block in the modern semiconductor industry, due to its flexibility and sharp heterointerface without atomic commensurability, by the vertically van der Waals bonding and absence of dangling bonds.^1^, ^2^, ^3^ Moreover, the adjustable band positions and bandgaps, which are dependent on the TMD type and number of layers, enable the artificial design of the functionalized band structure, i.e., the p–n junction and type‐II band structure in TMD‐based heterostructures (hetero‐TMDs).^4^ Until now, these hetero‐TMDs with functionalized band structures have attracted significant attention in a wide variety of applications, which include p–n diodes, photodetectors, and solar cells.^4^, ^5^, ^6^, ^7^, ^8^, ^9^, ^10^ Another emerging field for the application of hetero‐TMDs is that of the photo‐electrochemical (PEC) catalyst for hydrogen evolution, which is an environment‐friendly technology for the production of storable fuel using sustainable solar energy.

In PEC hydrogen evolution, p‐type silicon (p‐Si) has been widely used for photocathodes due to its narrow bandgap of 1.12 eV and suitable band position relative to the hydrogen redox potential. However, p‐Si photocathode has a low onset potential in electrolytes and poor kinetics for hydrogen ion adsorption at the surface. Hence, an additional catalyst is essential for the improvement of the p‐Si photocathode efficiency, and several studies have focused on the positive shift of the onset potential of p‐Si.^11^, ^12^, ^13^, ^14^, ^15^, ^16^, ^17^, ^18^ Among various catalysts, group 6 TMDs such as MoS_2_, WS_2_, and WSe_2_ were evaluated for their roles as promising hydrogen evolution reaction (HER) catalysts due to their proper band position, transparency, high specific surface‐to‐volume ratio, and high hydrogen adsorption potential.^15^, ^16^, ^17^, ^18^ Moreover, a sharp heterointerface between the TMDs and p‐Si allows for the minimization of the interfacial resistance and charge traps at the interface. In addition, the formation of the staggered gap (type‐II band) by an appropriate band alignment between the p‐Si photocathode and TMDs generates a built‐in electric field at the interface, which shifts the onset potential of p‐Si to more positive values.^15^ In particular, when a multiple of this staggered gap is formed (double‐staggered gap or multistaggered gap), the onset potential and photocurrent can be improved, given that a multistaggered gap facilitates the transfer of photogenerated electrons (holes) in PEC water splitting.^19^, ^20^ Hence, a significant improvement of the onset potential in the p‐Si photocathode can be realized through the deposition of a hetero‐TMD thin‐film catalyst with a multistaggered gap on the p‐Si photocathode. However, despite the significant potential of hetero‐TMDs as a catalyst for PEC hydrogen evolution, the challenging synthesis of large‐scale hetero‐TMDs limits its practical applications.

To date, various techniques have been implemented for the formation of hetero‐TMDs. The conventional direct synthesis via chemical vapor deposition (CVD) and the assembly technique (micromechanical stacking) yielded poor spatial uniformities and limited scales of hetero‐TMDs.^21^, ^22^, ^23^, ^24^, ^25^, ^26^, ^27^ Although the sulfurization of the sputter‐grown Mo/W thin films enables the formation of the large‐scale MoS_2_/WS_2_ heterostructure, this process is not capable of precise thickness control, and it is impossible to produce hetero‐TMD having different chalcogen compounds such as MoS_2_/WSe_2_.^28^, ^29^, ^30^ The high‐quality centimeter‐scale hetero‐TMD thin films were recently fabricated by dry transfer assembly using thermal release tape.^9^ However, this technique requires the individual deposition of each TMD for stacking, which is a significantly repetitive and complex process. Thus, such problems make it necessary to develop a straightforward in situ process for the formation of large‐scale hetero‐TMDs.

A suitable method for the fabrication of large‐scale hetero‐TMDs is pulsed laser deposition (PLD), which is a conventional in situ growth process for the fabrication of heterostructures and superlattice thin films.^31^, ^32^ In particular, the PLD method enables the fabrication of highly uniform large‐scale TMD thin films on various substrates, in addition to the precise control of the number of layers (*n*), which can be adjusted by the number of the laser pulses (*p*).^33^, ^34^, ^35^ Moreover, PLD has a fast deposition rate when compared with conventional methods.^35^ Hence, in this study, the PLD method was implemented for the fabrication of multi‐heterostructure TMDs (multi‐TMDs), and vertically stacked MoS_2_/WSe_2_ (MW′) and MoS_2_/WS_2_/WSe_2_ (MWW′) multi‐heterojunction structure thin films were successfully fabricated on a centimeter‐scale p‐Si substrate. The crystalline phase and uniformity of the PLD‐grown MWW′ thin film were characterized using Raman spectroscopy. To verify the staggered gap and real band position, ultraviolet–visible spectroscopy (UV–vis) and ultraviolet photoelectron spectroscopy (UPS) were employed. The multi‐TMD thin film with a multistaggered gap considering the bandgap and band position of each TMD significantly improved the PEC performance of the p‐Si photocathode as the hydrogen evolution catalyst. A discussion on the improved PEC performance of the p‐Si photocathode by the multi‐TMD thin‐film catalyst is presented in conjunction with the experimental results, i.e., linear sweep voltammetry (LSV) and electrochemical impedance spectroscopy (EIS) results.

## 2 Result and Discussion

**Figure 1:**
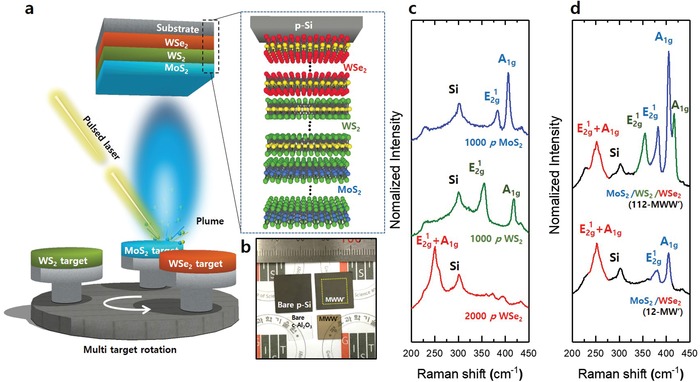
a) Schematic diagram of PLD method for fabrication of MWW′ thin film. b) Optical image that indicates the fabricated 112‐MWW′ thin films on the p‐Si and *c*‐Al_2_O_3_ substrates. c) Raman spectra for 1000 *p* MoS_2_, 1000 *p* WS_2_, and 2000 *p* WSe_2_ thin films on p‐Si. d) Raman spectra for 12‐MW′ and 112‐MWW′ thin films.

The MWW′ thin film was realized by the sequential deposition of WSe_2_, WS_2_, and MoS_2_ using PLD with a target rotation system (**Figure** **1**a). Figure 1b presents the optical images of the 1000 *p* MoS_2_/1000 *p* WS_2_/2000 *p* WSe_2_ (112‐MWW′) thin films on the centimeter‐scale p‐Si and the *c*‐sapphire (*c*‐Al_2_O_3_) substrate. Prior to the fabrication of the multi‐TMD thin films, the homostructure TMD (homo‐TMD) thin films were fabricated on a p‐Si substrate, to optimize the growth conditions, and the structure was characterized using Raman spectroscopy. Figure 1c presents the Raman spectra of the 1000 *p* MoS_2_, 1000 *p* WS_2_, and 2000 *p* WSe_2_ thin films. The E^1^_2g_ and A_1g_ peaks (main peaks) by the out‐of‐plane and in‐plane vibrations of the metal and chalcogens were observed at 383.5 and 405.1 cm^−1^ for 1000 *p* MoS_2_, and 354.5 and 418.3 cm^−1^ for 1000 *p* WS_2_. The Raman spectrum for the 2000 *p* WSe_2_ thin film exhibited the typical overlapped main peak (E^1^_2g_ + A_1g_) at 250.3 cm^−1^. In addition, the homo‐TMD thin films were fabricated with different values of *p* (1000 *p*, 2000 *p*, and 4000 *p*) for the control of thickness, and Raman spectroscopy was conducted to characterize their phases (Figure S1, Supporting Information). As displayed in Figure S1 (Supporting Information), the Raman spectra of the thickness‐controlled homo‐TMD thin films on p‐Si exhibit their intact main peaks.

Based on the deposition conditions of the homo‐TMD thin film, two types of multi‐TMD thin films were fabricated. Figure 1d presents the Raman spectra of the 1000 *p* MoS_2_/2000 *p* WSe_2_ (12‐MW′) and 112‐MWW′ thin films. In these MW′ and MWW′ Raman spectra, only the main peaks caused by the respective TMDs were observed. Moreover, there were no the mixed phases such as WS_2_ *_x_*Se_2−2_ *_x_* or M*_x_*W_1−_ *_x_*S_2_ in the Raman spectrum of the 112 MWW′ thin film, which indicates that each TMD layer may be appropriately deposited without an intermixing phase.^36^, ^37^ In this study, the homo‐ and multi‐TMD thin films were fabricated on p‐Si substrates, although the formation of TMD thin films on p‐Si substrates has been challenging using conventional methods such as CVD, due to the poor chemical affinity between the TMDs and Si, which has a hydrophilic surface.^15^

**Figure 2:**
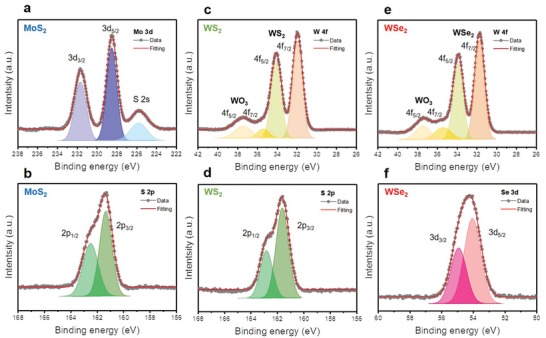
XPS spectra of a) Mo 3d and b) S 2p of the 1000 *p* MoS_2_ layer on p‐Si. c) W 4f and d) S 2p of the 1000 *p* WS_2_ layer on p‐Si. e) W 4f and f) Se 3d of the 2000 *p* WSe_2_ layer on p‐Si, respectively. The gray dots represent the experimental data, and the red line indicates the fitting results.

To verify the composition and chemical state of the individual elements of the PLD grown‐TMD thin film, X‐ray photoelectron spectroscopy (XPS) was conducted for each homo‐TMD thin film on p‐Si. **Figure** **2** presents the core‐level XPS spectra of Mo 3d (Figure 2a) and S 2p (Figure 2b) for the 1000 *p* MoS_2_ thin film, W 4f (Figure 2c) and S 2p (Figure 2d) for the 1000 *p* WS_2_ thin film, and W 4f (Figure 2e) and Se 3d (Figure 2f) for the 2000 p WSe_2_ thin film. All the XPS spectra were in good agreement with the typical XPS spectrum of each TMD.^35^, ^38^, ^39^, ^40^ As shown in Figure 2c,e, small doublet peaks at 35.4 and 37.5 cm^−1^ were observed due to the small portion of oxidized W (WO_3−_ *_x_*) or the domain edge.^40^ The calculated atomic ratios (chalcogen/metal) for the respective homo‐TMD thin films based on the fitted peak area were 2.05 for 1000 *p* MoS_2_, 2.05 for 1000 *p* WS_2_, and 2.09 for 2000 *p* WSe_2_. These atomic ratios are nearly equal to the ideal value of two, which indicates that the PLD‐grown TMD thin films are stoichiometric.

**Figure 3:**
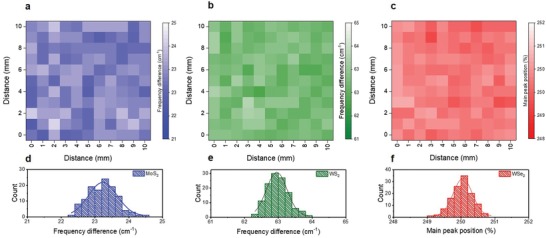
The Raman mapping based on ∆*f* values for a) the MoS_2_ layer and b) WS_2_ layer in the 112‐MWW′/p‐Si thin film. c) Main peak‐position‐based Raman mapping for the WSe_2_ layer in 112‐MWW′/p‐Si thin film. Histogram as a function of ∆*f* values for d) MoS_2_ and e) WS_2_ layer calculated from Raman maps in panels (a) and (b). f) Histogram as a function of the main peak position calculated from the Raman map in panel (c).

For practical applications, uniformity over the entire deposited film area is essential for TMD thin films. Thus, large‐scale (1 × 1 cm) Raman spectroscopy was conducted on the 112‐MWW′ thin film, to verify the spatial and thickness uniformity (**Figure** **3**). Figure 3a,b presents the Raman maps for MoS_2_ and WS_2_, which were dependent on the frequency difference between E^1^_2g_ and A_1g_ (∆*f*), given that the *n* value of the TMDs is affected by ∆*f*. Figure 3c presents the Raman map for WSe_2_ based on the main peak position, due to the overlapping main position. As shown in Figure 3a,b, the uniform ∆*f* values were observed in the Raman maps of MoS_2_ and WS_2_. Moreover, the Raman map of WSe_2_ exhibited a uniform peak position over the entire substrate area, as shown in Figure 3c. The above results suggest that the respective TMD layers were well deposited on the 1 × 1 cm^2^ scale without thickness gradation and a no‐growth region. To better understand the uniformity of the 112‐MWW′ thin films, histograms were plotted as a function of ∆*f* for the MoS_2_ (Figure 3d) and WS_2_ Raman maps (Figure 3e), and as a function of the main peak position for the WSe_2_ Raman map (Figure 3f). The center peak position of the Gaussian fitted histogram was 23.2 cm^−1^ for the MoS_2_ layer, 63.0 cm^−1^ for the WS_2_ layer, and 250.1 cm^−1^ for the WSe_2_ layer in the 112‐MWW′ thin films. The *n* value of MoS_2_ could be inferred using Equation (1), 34

(1) $$\Delta f=26.45-\frac{15.42}{1+1.44n^{0.9}}{\mathrm{cm}}^{-1}$$

Based on Equation (1), the calculated ∆*f* for the trilayer MoS_2_ was 23.3 cm^−1^. The result is consistent with the center peak position of ∆*f* (23.2 cm^−1^) for the 1000 *p* MoS_2_ Raman map. Thus, the MoS_2_ layer in the 112‐MWW′ thin film was estimated as a trilayer. In addition, the deviation of ∆*f* values was below ±1 cm^−1^, which indicates that MoS_2_ had a highly uniform thickness of three layers within the error range of ±1 layer on the entire deposited area. Moreover, WS_2_ was expected to have a uniform thickness within the error range of ±1 layer, given that the deviation of ∆*f* values for WS_2_ was also below ±1 cm^−1^. However, it was difficult to infer the correct thickness of WS_2_, given that the reported values of *n* dependent on ∆*f* were different.^41^, ^42^ In the case of WSe_2_, *n* could not be estimated using the main peak position due to peak overlapping. However, the uniform main peak position indicates that the WSe_2_ layer was highly uniform on the entire substrate area.^35^ Besides, the small deviation in the main peak intensity (below 10%) demonstrated the excellent thickness uniformity of WSe_2_ over the entire substrate area (Figure S2, Supporting Information). Consequently, the respective TMD layers were well stacked without no‐growth regions in the 112‐MWW′ thin films, and the 112‐MWW′ thin film exhibited an excellent thickness uniformity over the entire substrate area.

**Figure 4:**
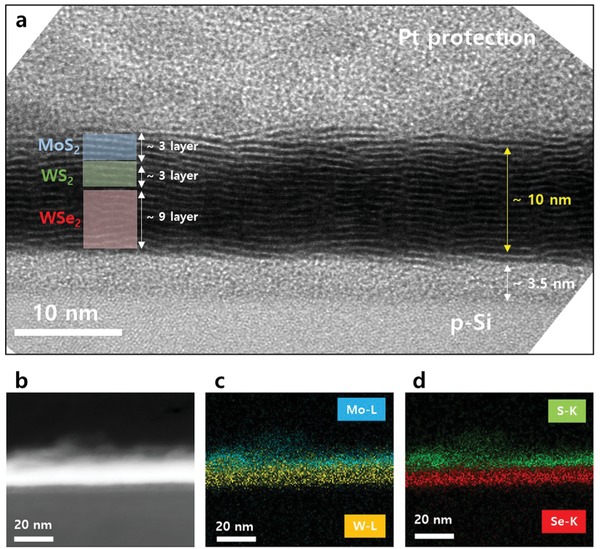
a) Cross‐sectional high‐resolution TEM image for 112‐MWW′/p‐Si thin film (total thickness of 112‐MWW′/p‐Si thin film was 10 nm, and that of the native oxide was ≈3.5 nm). This native oxide was formed during the deposition of TMDs although we removed native oxide using buffer oxide etchant (HF solution) before deposition of TMDs. b) STEM image of 112‐MWW′/p‐Si thin film. c) EDS mapping of Mo and W from the same area in panel (b). d) EDS mapping of S and Se from the same area in panel (b).

To verify the thickness of the 112‐MWW′ thin film, cross‐sectional transmission electron spectroscopy (TEM) analysis was conducted. The *n* of the 1000 *p* MoS_2_ thin film was estimated as three layers using Equation (1), and the *n* of the 2000 *p* WSe_2_ thin film was estimated as nine layers in the proposed system, when the previously reported empirical results were considered.^35^ **Figure** **4**a presents a cross‐sectional TEM image of the 112‐MWW′ thin film. The total thickness of the 112‐MWW′ thin film was 10 nm, and the thickness of each monolayer TMD was ≈0.62–0.67 Å. When the thickness of MoS_2_ and WSe_2_ were subtracted from the total thickness of 10 nm, the *n* of WS_2_ was estimated as three layers. However, the same crystalline structure made it difficult to distinguish each TMD layer in the TEM analysis results, as shown in Figure 4a. Thus, energy dispersive spectroscopy (EDS) mapping was further conducted on the 112‐MWW′ thin film. Figure 4b presents the cross‐sectional scanning‐TEM (STEM) image of the 112‐MWW′ thin film. From the EDS mapping for the L emission energies of Mo and W from the same area, as shown in Figure 4b, Mo and W were well distinguished (Figure 4c). In the EDS mapping for the K emission energies of S and Se, S and Se were well separated (Figure 4d).

**Figure 5:**
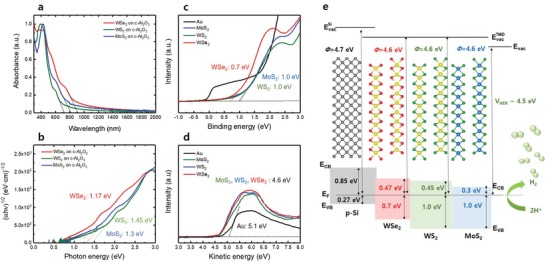
a) Normalized absorbance data for 1000 *p* MoS_2_, 1000 *p* WS_2_, and 2000 *p* WSe_2_ thin films on double polished *c*‐Al_2_O_3_ substrate. b) Tauc plot derived from absorbance data in panel (a). UPS data for determination of c) VBM as a function of binding energy and d) work function as a function of kinetic energy. e) Schematic diagram of energy band alignment of 112‐MWW′ thin film. *E*_CB_ represents conduction band minimum, and the bandgap of p‐Si was considered as 1.12 eV.

For the application of the fabricated multi‐TMD thin films (MW′ and MWW′) as HER catalysts, it is necessary to better understand the band alignment between p‐Si and multi‐TMDs. Theoretically, MWW′ and MW′ thin films have multistaggered gaps, considering the bandgap and band position of each TMD layer.^43^ To identify the real band structure, the work function, optical bandgap, and energy difference between the Fermi level (*E*_f_) and valence band maximum (*E*_VB_, VBM) were measured using UV–vis and UPS. **Figure** **5**a presents the absorbance graph for the 1000 *p* MoS_2_, 1000 *p* WS_2_, and 2000 *p* WSe_2_ thin films on *c*‐Al_2_O_3_ substrates. For an accurate estimation of the optical bandgap, a Tauc plot was derived from the absorbance graph for the respective TMD thin films, as shown in Figure 5b. The estimated optical bandgaps were 1.3 eV for 1000 *p* MoS_2_, 1.45 eV for 1000 *p* WS_2_, and 1.17 eV for 2000 *p* WSe_2_, respectively. The energy difference between *E*_f_ and *E*_VB_ (*E*_f_ − *E*_VB_) for the respective TMD thin films on the p‐Si photocathode was determined using UPS (Figure 5c). The measured *E*_F_ − *E*_VB_ was 0.7 eV for 2000 *p* WSe_2_/p‐Si, and 1.0 eV for both 1000 *p* MoS_2_/p‐Si and 1000 *p* WS_2_/p‐Si. The work functions were determined by the *x*‐intercepts of the extrapolated lines of kinetic energy, which is an energy of the secondary electron cut‐off subtracted from the incident photon energy of 21.2 eV. In addition, Au was used as a reference for more accurate work function measurements. As shown in Figure 5d, the work functions of each TMD thin film were equally observed as 4.6 eV. In the case of the bare p‐Si, the measured *E*_F_ − *E*_VB_ of the bare p‐Si was 0.27 eV, and the work function of p‐Si was 4.7 eV (Figure S3, Supporting Information).

Based on the measured results, a schematic of the energy‐band alignment for the homo‐TMD thin films (Figure S4, Supporting Information), 12‐MW′ thin film (Figure S5, Supporting Information), and 112‐MWW′ thin film (Figure 5e) was developed. As shown in Figure 5e and Figures S4 and S5 (Supporting Information), the homo‐TMDs and multi‐TMDs thin films exhibited single and multistaggered gaps, respectively, with the p‐Si photocathode. In general, the stepped‐down alignment of the conduction band minimum (CBM) and VBM between the photocathode and electrolyte facilitates the transfer of photogenerated electrons and holes due to the built‐in electric field of the staggered gap. Moreover, the small difference in the staggered gap expedites the electron transfer and minimizes the recombination of electrons and holes.^44^ Given that multi‐TMDs/p‐Si photocathodes have multistaggered gaps, multi‐TMD thin‐film catalysts can transfer photogenerated electrons more effectively than homo‐TMD thin‐film catalysts. In homo‐TMDs/p‐Si, WSe_2_ has the smallest gap difference with respect to p‐Si; thus, WSe_2_/p‐Si exhibits a more effective transfer of photogenerated electrons. The relatively higher work function of homo‐ and multi‐TMDs (4.6 eV) with than H^+^/H_2_ reduction potential, which is generally considered as 4.5 eV, enables the transfer of the photogenerated electrons to the electrolyte. Furthermore, the relatively negative valence band position of these TMD thin‐film catalysts with respect to p‐Si can block the hole transfer to the TMDs and electrolytes, thereby preventing hole recombination at the Si surface. Considering the band alignments as a whole, improved catalytic performances are expected from the TMD thin films on the p‐Si photocathode in the following order: MoS_2_ > WS_2_ > WSe_2_ > MW′ > MWW′.

**Figure 6:**
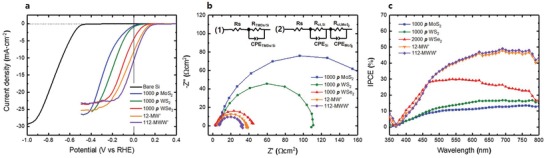
a) LSV plots, b) EIS spectra, and c) IPCE spectra of 1000 *p* MoS_2_, 1000 *p* WS_2_, 2000 *p* WSe_2_, 12‐MW′, and 112‐MWW′/p‐Si photocathodes in 0.5 m H_2_SO_4_ electrolyte. EIS and IPCE were measured at 0 V_RHE_. Equivalent circuit (1) in panel (b) was used for the fitting of the EIS data of the 1000 *p* WS_2_, 2000 *p* WSe_2_, 11 MW′, and 112 MWW′/p‐Si photocathodes. Equivalent circuit (2) was used for the fitting of the EIS data of the 1000 *p* MoS_2_/p‐Si photocathode.

To validate the practical PEC performances of the fabricated homo‐ and multi‐TMDs/p‐Si photocathodes, PEC measurements were carried out for the respective homo‐TMDs/p‐Si (1000 *p* MoS_2_, 1000 *p* WS_2_, and 2000 *p* WSe_2_) and multi‐TMDs/p‐Si (12‐MW′ and 112‐MWW′) using a standard three‐electrode cell in 0.5 m sulfuric acid. **Figure** **6**a presents the PEC photocurrent density as a function of the potential with respect to the reversible hydrogen electrode (RHE, V_RHE_) from the LSV analysis. The onset potential, which is defined as the potential at a photocurrent density of −1 mA cm^−2^, was positively shifted, and the photocurrent density at 0 V improved in the order of MoS_2_ > WS_2_ > WSe_2_ > MW′ > MWW′, as expected (**Table** **1**). This positive shift of the onset potential could lead to the effective production of hydrogen, and the rapid realization of the saturation photocurrent density with a relatively lower overpotential. Among the homo‐ and multi‐TMDs/p‐Si photocathodes, the 112‐MWW′/p‐Si exhibited the best photocurrent density of −11.45 mA cm^−2^ at 0 V_RHE_ in the proposed system. This photocurrent density satisfied the minimum photocurrent density (−8 mA cm^−2^) of the economically viable PEC cells.^45^

**Table 1:** PEC performance of TMDs/p‐Si thin films

PEC performance		
Photocathode	Onset potential [V]	Photocurrent density [mA cm^−2^]
1000 *p* MoS_2_/p‐Si	−0.02	−0.53
1000 *p* WS_2_/p‐Si	0.00	−0.92
2000 *p* WSe_2_/p‐Si	0.08	−4.38
12‐MW′/p‐Si	0.12	−8.05
112‐MWW′/p‐Si	0.14	−11.54

Although band structure of TMDs/p‐Si affects the PEC performance, the PEC performance can be influenced by intrinsic catalytic properties according to the type of TMDs. Considering the band structures of WSe_2_ and WS_2_ with the p‐Si photocathode, the PEC characteristics of WS_2_ and WSe_2_ should be similar, given that the CBM difference between WS_2_ and WSe_2_ is very small (0.02 eV). However, it is relatively more probable for the onset potential of WSe_2_ to shift to a more positive value than that of WS_2_, given that the catalytic properties of WSe_2_ for hydrogen ion adsorption are superior to those of WS_2_, according to the reported computational analysis.^46^ We also measured LSV curves for MoS_2_/p‐Si photocathodes dependent on *p* because onset potential can be shifted dependent on thickness of MoS_2_ (Figure S6, Supporting Information).^15^ As shown in Figure S6 (Supporting Information), there is no shift of onset potential in LSVs for MoS_2_/p‐Si photocathodes, indicating that there is no thickness effect in our TMD thin‐film catalyst.

In addition, although the TMD catalyst improved the onset potential of the p‐Si photocathode, the saturated photocurrent density of TMDs/p‐Si underwent a decrease than that of bare p‐Si, given that the amount of the light irradiated onto p‐Si was reduced by the semitransparent TMD catalysts. However, one interesting is that 12‐MW′/p‐Si photocathode shows higher saturated photocurrent density than 2000 *p* WSe_2_/p‐Si photocathodes, and 112‐MWW′/p‐Si photocathode shows similar saturated photocurrent density compared to 2000 *p* WSe_2_/p‐Si although the transmittance of both 12‐MW′ and 112‐MWW′ is lower than 2000 *p* WSe_2_. This is because the multistaggered gap of multi‐TMDs/p‐Si photocathodes enables not only the effective electron transfer but also the hole block rather than single staggered gap of WSe_2_/p‐Si photocathode.

To further understand the surface kinetics based on the TMD thin‐film catalysts, EIS measurements were carried out under AM 1.5 G illumination at 0 V_RHE_. The impedance spectral analysis was useful to better understand the electrochemical interface reaction. Figure 6b presents the Nyquist plots of the EIS spectra for the respective TMD thin‐film catalysts. The Nyquist plots of the TMD thin‐film catalysts, with the exception of MoS_2_, were displayed as a single semicircle and fitted with an equivalent Randles circuit; which consisted of constant phase elements (CPEs) associated with the TMDs/p‐Si (CPE_TMDs/Si_), and charge‐transfer resistances (*R*_ct_) from the TMDs/p‐Si to the electrolyte (*R*_ct,TMDs/si_), as displayed by circuit (1) (Figure 6b). The Nyquist plot of the MoS_2_ thin film on the p‐Si displays two distinguishable semicircles (Figure S7, Supporting Information), and these data were fitted using a general equivalent equation of a thin‐film catalyst–semiconductor system, which consisted of CPEs associated with the p‐Si (CPE_Si_) and MoS_2_ (CPE_MoS2_), and *R*_ct_ from p‐Si to MoS_2_ (*R*_ct,Si_) and from MoS_2_ to the electrolyte (*R*_ct,MoS2_), as displayed by circuit (2) (Figure 6b). *R*_S_ represents the overall series resistance, which includes the resistance between the contact metal and p‐Si and the solution resistance, among others.

According to the fitted data, as shown in **Table** **2**, the *R*_S_ values of all the photocathodes were similar, which indicates that the *R*_S_ values of the homo‐ and multi‐TMD thin films were constant. In particular, the *R*_ct,Si_ was significantly low (4.5 Ω cm^2^) in the Nyquist plot of the MoS_2_ catalyst, and the other homo‐ and multi‐junction TMD thin‐film catalysts exhibited single semicircles in the Nyquist plots, although they possessed multiple interfaces. These results indicate that the interface resistance between the TMDs and Si was considerably small, due to the proper band position and significantly sharp heterointerface between the TMDs and Si without dangling bonds and with vertical van der Waals bonding. Hence, the photogenerated electrons could be effectively transferred from Si to the TMDs.

**Table 2:** Charge‐transfer resistance with respect to the TMDs/p‐Si thin films

Charge‐transfer resistance		
Photocathode	*R*_S_/*R*_ct,Si_ [Ω cm^2^]	*R*_ct,TMDs_ [Ω cm^2^]
1000 *p* MoS_2_/p‐Si	3/4.5	193
1000 *p* WS_2_/p‐Si	6/–	106
2000 *p* WSe_2_/p‐Si	4/–	40
12‐MW′/p‐Si	5.5/–	31.5
112‐MWW′/p‐Si	7.2/–	25

Furthermore, it was confirmed that the transfer of photogenerated electrons from TMDs/p‐Si to the electrolyte was improved in the order of MoS_2_ > WS_2_ > WSe_2_ > MW′ > MWW′, given that *R*_ct,TMDs_ decreased in the order of MoS_2_ > WS_2_ > WSe_2_ > MW′ > MWW′, which corresponds to the band alignment and the LSV results. It should be noted that *R*_ct,MWW′_ was found to be significantly smaller than *R*_ct,MoS2_, although the top surfaces of both films were of the same material as the vertically stacked MoS_2_, which indicates that the catalytic properties of both films for hydrogen ion adsorption were the same. This further indicates that the multistaggered gap in 112‐MWW′/p‐Si reduced *R*_ct_ significantly more than that of MoS_2_/p‐Si, which had a single‐staggered gap. Consequently, the multistaggered gap of the TMDs improved the PEC performance of the p‐Si photocathode.

Among various efficiency models for the evaluation of the conversion efficiency of solar energy to hydrogen, incident‐photon‐to‐current conversion efficiency (IPCE) is a useful approach for the determination of the device efficiency based on the incident photon as a function of the illumination wavelength. To verify the efficiency of the respective TMD thin‐film catalysts, the IPCE was measured for the respective TMDs/p‐Si thin films at 0 V_RHE_ using the following equation

(2) $$\mathrm{IPCE}\left(\%\right)=\frac{j\left(A\;{\mathrm{cm}}^{-2}\right)\times 1240}{\lambda \left(\mathrm{nm}\right)\cdot I_{\mathrm{inc}}\left(W\;{\mathrm{cm}}^{-2}\right)}\times 100$$

where *j* is the photocurrent density, and *I*_inc_ is the power intensity at a specific wavelength (λ). The multi‐TMDs/p‐Si thin films exhibited a superior efficiency to the homo‐TMDs/p‐Si thin films over the entire spectral range, and the efficiency increased within the visible light region in the order of MoS_2_ > WS_2_ > WSe_2_ > MW′ > MWW′, which corresponds to the band alignment trend, in addition to the LSV and EIS results.

Additionally, the chronoamperometric measurement was carried out at an applied potential of −0.2 V_RHE_ under AM 1.5 G to confirm the stability of multi‐TMDs/p‐Si photocathodes. The photocurrent density of both multi‐TMDs/p‐Si photocathodes displays the significantly stable photocurrent density as over 80% of the initial photocurrent density for 12 h (Figure S8, Supporting Information). To further confirm whether the measured photocurrent density is caused by hydrogen evolution, we measure the Faradaic efficiency for MWW′ photocathode (Figure S9, Supporting Information). As shown in Figure S9 (Supporting Information), the average H_2_‐Faradaic efficiency is 84.3%, indicating that photogenerated electrons are mostly used to convert hydrogen.

The measured PEC characteristics suggest that the MWW′ thin‐film catalyst with the multistaggered gap significantly improves the performance of PEC hydrogen evolution. In particular, the vertically well‐stacked multijunction structure without other structures such as edge‐side TMD helped clarify the effect of multistaggered gaps on the PEC performance of TMD catalysts. Moreover, there are still ample rooms to improve the PEC performance. First, the combination of the edge sites of the TMDs and multi‐TMDs can improve PEC performance, given that the edge sites of TMDs have excellent catalytic performances.^15^, ^47^ Also, native oxide is one of the obstacles to the effective transfer of photogenerated electrons from Si to the electrolyte.^48^ Thus, if the native oxide is perfectly removed between p‐Si and multi‐TMDs, the PEC performance of multi‐TMDs/p‐Si will be very improved.

## 3 Conclusion

In conclusion, large‐scale multijunction structured TMD thin films such as MW′ and MWW′ were successfully fabricated using PLD, which was proven to be a very effective approach for the fabrication of multi‐TMD thin films. The PLD‐grown MWW′ thin film exhibited excellent thickness uniformity and superior spatial uniformity without a no‐growth region, even on the hydrophilic Si substrate. Moreover, the MWW′ thin film had a multistaggered gap like the originally designed thin film. The PLD‐grown MWW′ thin films were additionally applied as an HER catalyst of the p‐Si photocathode among various possible fields, and it was also demonstrated that multi‐TMD thin‐film catalysts significantly improve the charge‐transfer performance when compared with homo‐TMD thin‐film catalysts, due to the multistaggered gap. Furthermore, the fabrication of various TMD‐stacked structures is expected to enable the development of new types of multi‐TMDs such as superlattices, which can be implemented in a wide variety of applications.

## 4 Experimental Section

*Fabrication of TMD Thin Films*: Commercial TMD targets (LTS Research Laboratories, Inc.) were used, in addition to the stoichiometric MoS_2_ and WS_2_ targets. Moreover, a Se supersaturated WSe_2.2_ target was used to prevent Se deficiency. The boron‐doped p‐Si substrate was used (Prime grade), and the resistivity of Si was 1–10 Ω. The Si substrate was cleaned using acetone, methanol, and isopropyl alcohol to remove organic particles, and it was etched in a buffer oxide etchant solution to remove the native oxide layer. The PLD growth conditions were as follows: the substrate temperature, energy density, and repetition were 500 °C, 1 J cm^−2^, and 3 Hz, respectively; and an Ar buffer gas was used to reduce the splashing effect and prevent the chalcogen deficiency during the film growth. The base pressure remained below 5.0 × 10^−7^ Torr, and the working pressure was set as 100 mTorr. The thickness was controlled by *p*, and *p* was used to indicate the thickness of the TMD thin film. For example, 1000 *p* MoS_2_ indicates that the irradiated total *p* was 1000 during the MoS_2_ deposition using PLD. The thicknesses of the respective homo‐TMD (2000 *p* for WSe_2_, 1000 *p* for MoS_2_ and WS_2_) thin films were the minimum thicknesses at which degradation was not observed during the PEC measurement.

*Structure Characterization of the Multi‐TMD Thin Film*: The structural characterization was estimated using Raman spectroscopy with a 514.5 nm laser. The composition, work function, VBM, and chemical bonding state of the TMD thin films were characterized using multipurpose XPS with UPS (Thermo VG Scientific). The thickness and layered structure were verified using a high‐resolution TEM measurement with EDS analysis (Tecnai G2 F30 S‐Twin). The Tacu plot used for the bandgap analysis was (*αhν*)^1/2^ = *A* (*hν* − *E*_g_), where α is the absorption coefficient, *hv* is the photon energy, *E*_g_ is the optical bandgap, and *A* is the absorbance.

*Fabrication of PEC Cell and PEC Measurement*: The Si backside was scratched using a diamond pencil, for the formation of a good contact between the metal and Si backside. The Cu wire was attached onto the Si backside by the In–Ga eutectic alloy and silver paste. PEC cell was covered except for TMDs/p‐Si part using an epoxy resin to passivate PEC cell from the electrolyte. Moreover, PEC measurements were carried out under AM 1.5 G illumination (100 mW cm^−2^) using a 150 W xenon lamp (Model 10 500, ABET Technology). The used reference electrode was a saturated calomel electrode, and Pt wire was used for the counter electrode. For the LSV measurements, the scan rate was 20 mV s^−1^ at 10 mV intervals. The EIS measurements were carried out from 0.1–100 000 Hz at 0 V_RHE_. For the IPCE measurement, a monochromator (Mmac 200, Dongwoo OPTRON) was used, and the IPCE was also measured at 0 V_RHE_. Stability of multi‐TMDs/p‐Si photocathodes was analyzed using chronoamperometric measurement at −0.2 V_RHE_ under AM 1.5 G. For stability measurement, a graphite rod was used instead of Pt counter electrode to avoid Pt deposition on the photocathode, and the electrolyte was stirred using a magnetic stirrer to remove the hydrogen bubble onto photocathode. For the measurement of Faradaic efficiency, the gas chromatography system (Agilent Tech 7820A, 5 A° molecular sieve column) was used with a thermal conductivity detector.

## Conflict of Interest

The authors declare no conflict of interest.

## Supporting information

Supplementary
