# Supplementary material for: Direct In Situ Growth of Centimeter‐Scale Multi‐Heterojunction MoS2/WS2/WSe2 Thin‐Film Catalyst for Photo‐Electrochemical Hydrogen Evolution
Source: Adv Sci (Weinh). 2019 Apr 26;6(13):1900301. doi: 10.1002/advs.201900301 (PMC6662091; doi:10.1002/advs.201900301)
Supplement: Supplementary file 1 — Supplementary [file ADVS-6-1900301-s001.pdf]

Copyright WILEY-VCH Verlag GmbH & Co. KGaA, 69469 Weinheim, Germany, 2019.

Supporting Information

for *Adv. Sci.,* DOI: 10.1002/advs.201900301

Direct In Situ Growth of Centimeter-Scale Multi-Heterojunction $MoS_2/WS_2/WSe_2$ Thin-Film Catalyst for Photo-Electrochemical Hydrogen Evolution

*Sehun Seo, Seungkyu Kim, Hojoong Choi, Jongmin Lee, Hongji Yoon, Guangxia Piao, Jun-Cheol Park, Yoonsung Jung, Jaesun Song, Sang Yun Jeong, Hyunwoong Park, and Sanghan Lee**

Copyright WILEY-VCH Verlag GmbH & Co. KGaA, 69469 Weinheim, Germany, 2019.

Supporting Information

Direct In situ Growth of Centimeter-scale Multi-heterostructure $MoS_2/WS_2/WSe_2$ Thin Film for Photoelectrochemical Hydrogen Evolution Catalyst

*Sehun Seo, Seungkyu Kim, Hojoong Choi, Jongmin Lee, Hongji Yoon, Guangxia Piao, Jun-Cheol Park, Yoonsung Jung, Jaesun Song, Sang Yun Jeong, Hyunwoong Park, and Sanghan Lee**

S1. Raman spectra for homo-TMD thin film with respect to ***p***

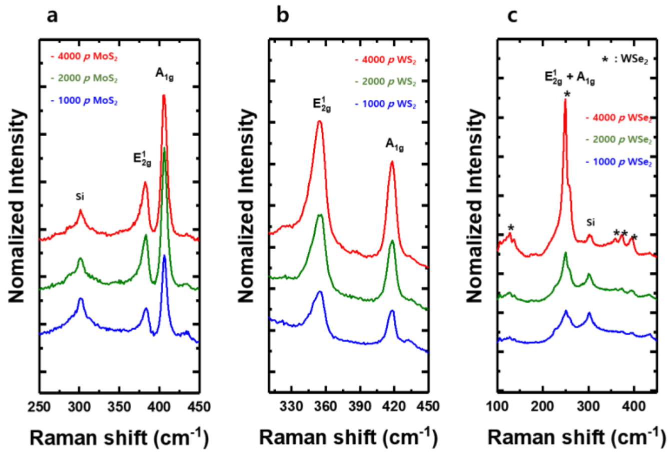

Figure S1. Raman spectra of a) MoS_2 thin films on p-Si, b) WS_2 thin films on p-Si, and c) WSe_2 thin films on p-Si with different ***p*** (1000 ***p***, 2000 ***p***, and 4000 ***p***). Star (*) indicates the Raman peak which is caused by the WSe_2.

S2. Raman mapping for WSe_2 layer in 112-MWW' thin film

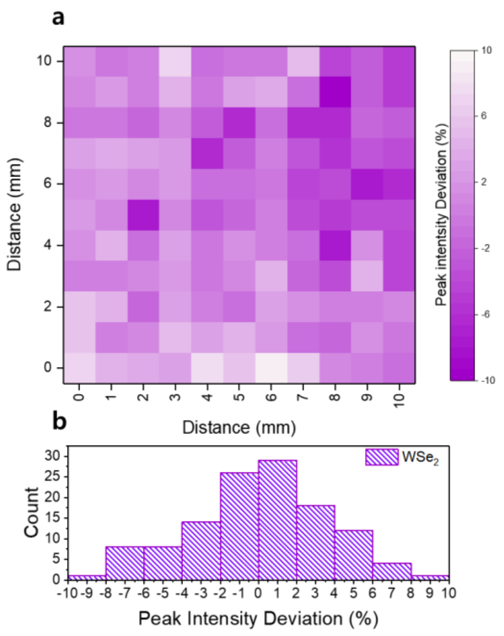

Figure S2. a) Raman mapping based on peak intensity deviation of the main peak for the WSe_2 layer in the 112-MWW' thin film; and b) histogram as a function of main peak intensity deviation which is calculated from the Raman map in Figure S2a.

S3. UPS data of p-Si

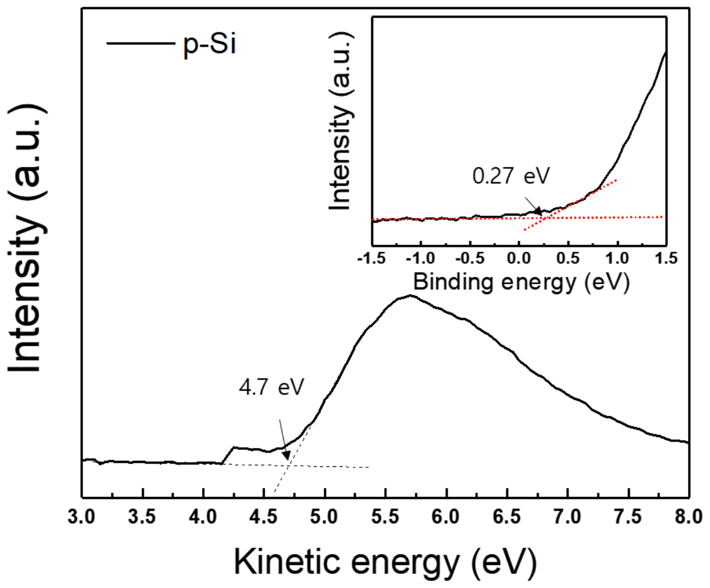

Figure S3. UPS data for determination of work function as a function of kinetic energy and the valence band maximum as a function of binding energy (inset) for p-Si photocathode.

S4. Band alignment of homo-TMD/p-Si thin films.

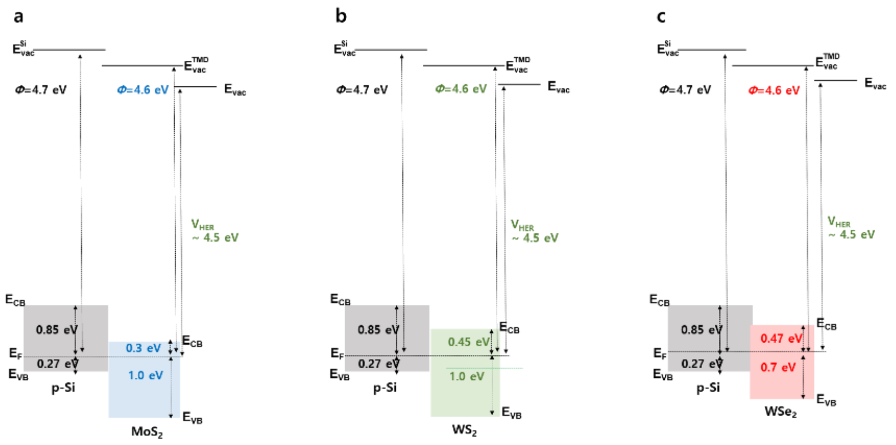

Figure S4. Schematic diagram of energy band alignment of a) 1000 ***p*** MoS_2/p-Si, b) 1000 ***p*** WS_2/p-Si, and c) 2000 ***p*** WSe_2/p-Si thin films.

S5. Band alignment of 12-MW'/p-Si thin film.

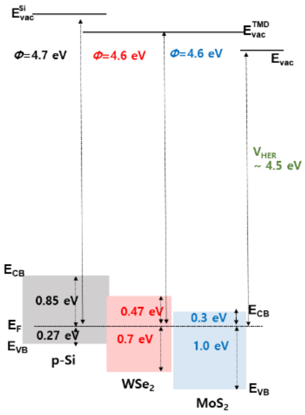

Figure S5. Schematic diagram of energy band alignment of 12-MW'/p-Si thin film.

S6. LSV curves for MoS_2/p-Si photocathodes

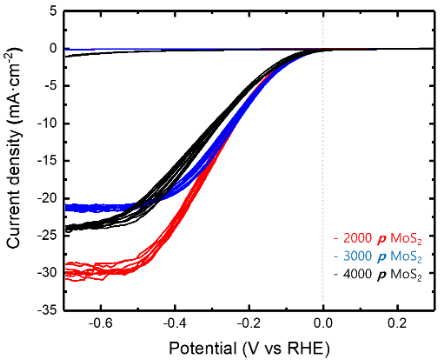

Figure S6. LSV plots for 2000 ***p***, 3000 ***p***, and 4000 ***p*** MoS_2/p-Si photocathodes in 0.5 M H_2SO_4 electrolyte. In LSV result for 4000 ***p*** MoS_2/p-Si photocathode shows dark-current of ~1 mA·cm^{-2}, and hence the photocurrent of 4000 ***p*** MoS_2/p-Si photocathode is higher than that of 3000 ***p*** MoS_2/p-Si photocathode.

S7. EIS spectra of 1000 ***p*** MoS_2

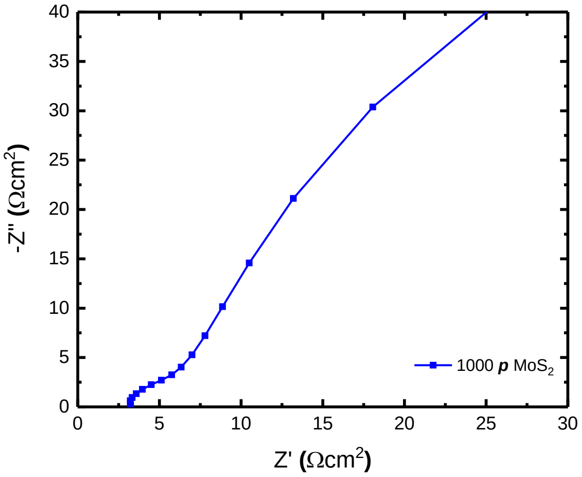

Figure S7. EIS spectrum of 1000 ***p*** MoS_2/p-Si photocathode. Two distinguishable semicircles were observed in the Nyquist plot of the 1000 ***p*** MoS_2/p-Si photocathode.

S8. Stability measurement for 112-MWW’/p-Si photocathode.

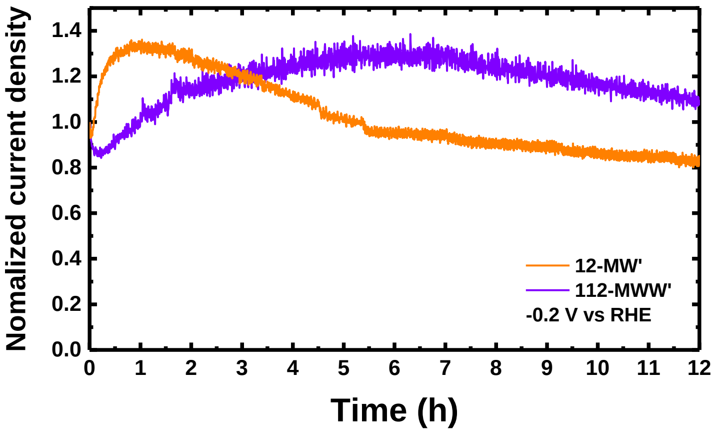

Figure S8. Stability measurement for both 12-MW’/p-Si and 112-MWW’/p-Si photocathodes.

S9. Faradaic efficiency for 112-MWW'/p-Si photocahtode

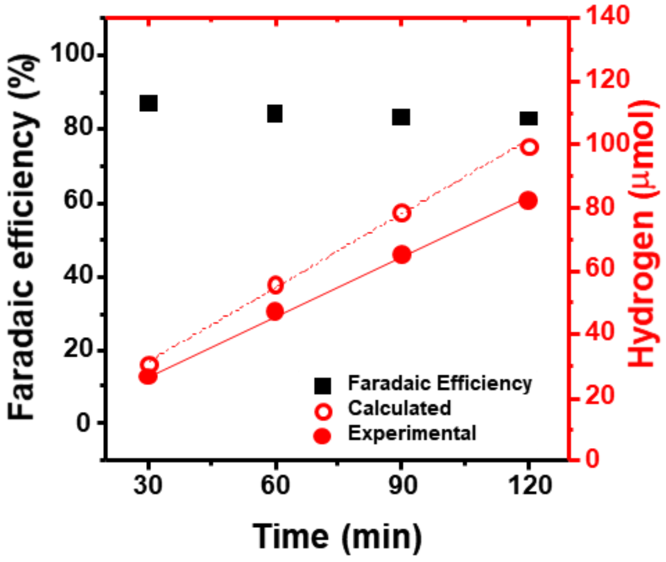

Figure S9. Faradaic efficiency and hydrogen production at -0.2 V_{RHE} under illumination for 112-MWW'/p-Si photocathode. The black solid square indicates the faradaic efficiency. The red open circle represents the calculated amount of ideal hydrogen production. The red solid circle represents the experimental amount of actual hydrogen production.
